# Development of a new cellular immunological detection method for tuberculosis based on HupB protein induced IL-6 release test

**DOI:** 10.3389/fmicb.2023.1148503

**Published:** 2023-04-03

**Authors:** Xiaoli Yu, Shengsheng Jiang, Yang Li, Huaiheng Zhou, Yutong Wei, Xuefang Li, Ye Zhang, Peng Hu, Haoming Wu, Hualin Wang, Shimin Wu, Shulin Zhang

**Affiliations:** ^1^School of Life Science and Technology, Wuhan Polytechnic University, Wuhan, China; ^2^Center for Clinical Laboratory, General Hospital of the Yangtze River Shipping, Wuhan Brain Hospital, Wuhan, China; ^3^Department of Immunology and Microbiology, Shanghai Jiao Tong University School of Medicine, Shanghai, China

**Keywords:** tuberculosis, HupB, IL-6, PBMCs, IGRA

## Abstract

**Objective:**

As a virulence factor, HupB plays important roles in the survival of MTB after infection and modulates the host immune response. In the current study, we aim to explore a new cellular immunological detection method for tuberculosis infection detection based on HupB protein.

**Methods:**

HupB was used to stimulate PBMCs extracted from pulmonary tuberculosis (PTB) patients, and secreted cytokines was examined. Then, we constructed a single center and a multi-center clinical trials to collect PBMCs from PTB patients, nPTB patients, or healthy volunteers to verify our findings.

**Results:**

Cytokine’s screening illustrated that IL-6 was the only cytokine released after HupB stimulation. Single-center and multi-center clinical trials showed that HupB stimulation significantly increased the level of IL-6 in the supernatant of PBMCs from PTB patients. Then we compared the specificity and sensitivity of HupB induced IL-6 release assay with ESAT-6 and CFP10 induced interferon γ release assay (IGRA), and found in smear positive PTB patients, the specificity and sensitivity of HupB induced IL-6 release assay was better than IGRA, and in smear negative PTB patients, the sensitivity was better. Combination of both assays provided an improved specificity and sensitivity for tuberculosis diagnosis.

**Conclusion:**

This study explored an immunological detection method for tuberculosis infection cells based on HupB protein-induced IL-6 release test, which can be used to enhance the diagnosis diagnostic accuracy of TB.

## Introduction

Tuberculosis (TB) is a contagious infectious disease caused by *Mycobacterium tuberculosis* (Mtb), mainly affects the lungs but can also affect any other organ including bone, brain and intestine etc. The latest global TB report published by World Health Organization (WHO) indicated that in 2022 (World Health Organization [WHO], 2022), almost 10.6 million people developed active TB disease, and 1.6 million TB deaths. TB was the top infectious disease cause of mortality globally, only except 2020, which COVID-19 pandemic was the top one.

By 2030, the WHO plans to reduce annual TB cases per 100,000 people by 80% from 2015 levels; The annual number of TB deaths has fallen by 90 percent from 2015 (World Health Organization [WHO], 2022). Early diagnosis and treatment of TB play the crucial roles to achieve the goal (Dheda et al., 2016). The TB test tools included bacteriological test like sputum smear or sputum culture test; imaging tests based on chest X-ray or CT scan; molecular biological test and immune test However, all these tests had respective limitations the imaging test was hard to distinguish TB and other lung diseases like pneumonia induced shadow, and lung cancer or pulmonary nodule. Moreover, imaging tests cannot differentiate between forepassed and current infection (Joo et al., 2021). In addition to bacteriological methods, imaging methods, molecular biology diagnosis and immunological diagnosis methods, respiratory tube endoscopy can detect tuberculosis lesions. A combination of the patient’s clinical history and the above diagnostic methods will have a good effect on the diagnosis of tuberculosis. Although sputum smear can identify activated TB quickly, but many patients cannot provide sputum samples for smear tests. The detectable rate was lower than 30% (false positive, lower sensitivity), hard to be used in children with TB or extra pulmonary tuberculosis diagnosis, it is hard to be used in children TB means getting a sputum sample from a child is not very convenient; the sputum culture test was the golden standard for TB diagnosis, but cost 3–4 weeks to get the results, was not meet the requirement of early diagnosis (Rodríguez-Hernández et al., 2020; Yang et al., 2020). Molecular biology testing is fast and time-saving, but requires expensive equipment and testing personnel with high technical knowledge, but has the weakness to distinguish dead or active Mtb (Rozales et al., 2014). So immune tests were needed for TB early diagnosis.

Among the immune tests, the Tuberculin skin test (TST) and interferon-γ release assay (IGRA) were widely used in TB diagnosis, TST was the traditional cell-mediated immune test, which can get the results in 2–3 days, but the sensitivity was limited (Auld et al., 2013; Haas and Belknap, 2019). The disadvantage is that this method is more likely to produce false negative results for tuberculosis patients such as children with tuberculosis, elderly patients with tuberculosis, and tuberculosis patients with immune dysfunction. Due to the high vaccination rate of BCG in our country, there is a high false positive rate in the TST skin test, and the reliability of its diagnosis is worth discussing. IGRA examines the released IFN-γ level from T cells stimulated by Mtb antigens (ESAT-6 and CFP-10, TB7.7 or CD8 **+** T cell antigen) using ELISA or enzyme-linked immunosorbent (Theel et al., 2018; Shafeque et al., 2020). The typical IGRA kits include QFT-G (Quantiferon TB Gold), QuantiFERON-TB Gold In-Tube (QFT-GIT), QuantiFERON-TB Gold Plus (QFT-Plus) and T-SPOT (Takeda et al., 2020; Won et al., 2020). However, IGRA cannot differentiate active TB or latent TB, and has relatively high false-negative rate (Awoniyi et al., 2016; Wu et al., 2017). Tuberculosis Infection T Cell Test (T-SPOT): Also known as gamma interferon-releasing specific T cell detection. T-spot is a T-cell immunity SPOT test designed by using tuberculous effector T lymphocytes in peripheral blood mononuclear cells of infected tuberculosis patients to secrete IFN-γ after stimulated by the specific antigen of tuberculosis bacteria (ESTA-6, CFP-10). By counting the number of T cells secreting IFN-γ, it has high sensitivity and specificity to identify tuberculosis infection or pathogenicity. If the test results are negative, the basic can exclude tuberculosis infection. If the result is positive, you may be currently infected with TB, or you may have been infected in the past, and the body’s immune cells retain the memory (Ma et al., 2019). Qft-plus is based on an improved version of the QuantiFERON-TB Gold In-Tube (QFT), which stands for Mycobacterium tuberculosis infection diagnostic kit for gamma-interferon release assay, also known as TB infection T cell assay. Tuberculobacter patients have specific T lymphocytes that secrete gamma-interferon when they are stimulated again by the specific antigen of the TB bacterium. By measuring the level of gamma-interferon released by cells in the blood, it can help detect the presence of TB infection. Gamma-interferon release tests are more sensitive and specific than the tuberculin test and are not affected by BCG and most non-tuberculous mycobacteria (Shafeque et al., 2020). It is desiderated to develop some new tools for TB diagnosis, particularly for HIV-positive TB people. Tuberculosis is a serious threat to human health, and among all kinds of opportunistic infections, tuberculosis is the most common co-infection of AIDS. Early TB-related screening, diagnosis and early treatment of HIV infected persons can further control the emergence of Mycobacterium tuberculosis infection in HIV infected persons. The clinical symptoms of TB in HIV/TB co-infected patients, including cough, phlegm, fever, weight loss, and night sweats, are atypical, which can lead to low immunity and increased risk of death. The combined diagnosis of various examinations is helpful for the early diagnosis and treatment of HIV/TB patients, so that the patients with HIV/TB co-infection can be better controlled.

In our previous study, we successfully identified a secretory protein from the culture filtrate of H37Rv standard strain using biomimetic affinity technology, HupB (Rv2986c) (Ma et al., 2017). As a nucleus-associated DNA binding protein, HupB is a homolog of HU in *Mycobacterium*. HU protein is one of the most conserved and abundant nucleosomes-associated proteins (NAPs) in bacteria, responses for maintaining the highly organized and dynamic structure of the chromosome, plays the roles similar as histones in eukaryote (Gupta et al., 2014; Kriel et al., 2018). In mycobacterium, HupB is involved in chromosome replication and transcriptional regulation, and combines with DNA to protect DNA. Moreover, some studies indicated that HupB has the ferroxidase activity in *Mycobacterium Bovis* and *Mycobacterium* leprae, and regulates the balance of iron and ferrous ions through the Fenton reaction to avoid the toxic effect of iron ions. So HupB is a virulence factor that affects the survival of MTB (Takatsuka et al., 2011).

On the other hand, HupB has immunomodulatory effects on the host and has the potential to enhance T cells against MDR-TB (Hadizadeh Tasbiti et al., 2016). In addition, a study by Van et al. showed that HupB induced and enhanced the killing effect of CD4 **+** T cells on pathogenic bacteria through methylation modification (Hadizadeh Tasbiti et al., 2016).

In the current study, we aimed to explore the potential of HupB as an Mtb antigen for TB diagnosis. We found that HupB stimulation induced IL-6 release in immune cells *in vitro*. We then collected clinical blood samples of TB people from six hospitals in China to evaluate the ability of HupB for TB diagnosis.

## Materials and methods

### Selection criteria for clinical diagnosis of pulmonary tuberculosis

To explore and further validate a reliable and effective cell immune response-based tool for TB diagnosis, clinical participants aged 18–65 years were grouped according to diagnostic criteria for tuberculosis issued by the Ministry of Health of China and international tuberculosis control regulations revised by WHO:

A. Bacteriological positive: positive sputum smears, positive molecular detections, positive T-SPOT results positive IGRA results.

B. Bacteriological negative: three consecutive negative sputum smears, and a negative sputum culture (negative T-SPOT results or negative IGRA results was recommended) and has three items in (1)–(5) or any of the following (6)–(7) can be diagnosed:

(1) Typical TB clinical symptoms and chest radiography. (2) Anti-TB treatment was effective. (3) Other non-tuberculous pulmonary diseases may be excluded clinically. (4) Pure protein derivatives (PDD) (5 units) strong positive, positive serology for anti-TB. (5) positive PCR + DNA probe detection of TB results. (6) Acid-resistant *Mycobacterium* was detected in Bronchoalveolar lavage (BALF). (7) Bronchial or pulmonary histopathology confirmed tuberculosis.

C. Non-tuberculous pulmonary diseases: non-tuberculous pulmonary infection, lung cancer, chronic obstructive pulmonary disease, and emphysema.

D. Healthy people (no clinical symptoms of diseases, participants with the following conditions were excluded from previous heart, lung, liver, spleen, blood, immune and metabolic system diseases. And require blood routine and urine routine to be within the normal range).

### Sample collection and information registration

All participants were recruited from three hospitals in Wuhan (Wuhan Jinyintan hospital, Wuhan Polytechnic University Hospital, General Hospital of the Yangtze River Shipping), Henan Provincial Chest Hospital, Xiangyang city hospital for tuberculosis and Xiangyang Central Hospital, and other six hospitals. A total of 986 cases were collected and 784 cases were enrolled (202 cases who did not meet the selection criteria for clinical diagnosis of pulmonary tuberculosis in 1.1 were excluded), including pulmonary tuberculosis (PTB) group and non-tuberculous pulmonary disease patients (nPTB) groups and Health control (HC) groups. Fresh blood samples were stored in an anticoagulant tube and isolation of peripheral blood mononuclear cells were within 24 h. Among them, the experimental group data statistics are shown in Table 1.

**TABLE 1:** Experimental sample grouping data statistics.

Classification	Age (mean value)	Quantity	Gender (male/female)
**1. Analysis of cytokines secretion from HupB stimulated PBMCs**			
PTB	18–56 (37)	11	9/2
HC	22–34 (28)	12	9/3
Total	18–65 (40)	23	18/5
**2. HupB was the only known antigen to stimulate IL-6 secretion in PBMCs from PTB patients**			
PTB	19–65 (45)	64	44/20
**3. Single-center small sample clinical validation of HupB stimulated IL-6 releasing in PBMCs**			
PTB	18–65 (41)	49	29/20
nPTB	20–73 (50)	23	14/9
HC	19–47 (31)	40	29/11
Total	18–73 (41)	112	72/40
**4. Multi-center trial for HupB induced IL-6 release assay**			
PTB	18–65 (48)	190	127/63
nPTB	21–28 (35)	33	21/11
HC	18–25 (23)	273	145/128
Total	18–65 (36)	496	292/204
**5. Comparison of HupB induced IL-6 release assay and IGRA for TB diagnosis**			
Classification	Age (mean value)	Quantity	Gender (male/female)
SP PTB	19–68 (48)	28	18/10
SN PTB	26–69 (50)	25	17/8
HC	20–65 (27)	36	19/17
Total	19–69 (42)	89	54/35

PBMCs, peripheral blood mononuclear cells; PTB, tuberculosis group; nPTB, non-tuberculous pulmonary disease patients; HC, healthy group; SP PTB, smear positive PTB group; SN PTB, smear negative PTB group; Total, All enrollees.

The registration form for blood sample sources collected included: the date of blood sample collection, patient data (basic information, hospitalization number, clinical symptoms, doctor diagnosis results, tuberculosis diagnosis technology results), healthy group (basic information, physical examination project information: 1. Hepatitis B and C HIV syphilis quantification; 2. Urine routine; 3. Whole blood cell count; and 4. Four coagulation items).

### Isolation of peripheral blood mononuclear cells

A total of 2 ml fresh whole blood was obtained and diluted with an equal volume of sterile PBS (Hyclone). Peripheral blood mononuclear cell (PBMC) were isolated according to the instructions of the peripheral blood mononuclear cell separation reagent (Sigma-Aldrich). Cell density was adjusted to 5 × 10^5^ cells/ml by using RPMI 1640 complete culture medium and counting by cell counter. The cell suspension was added to the 12-well plate at 2 ml/well and cultured in a cell incubator at 5% CO_2_ and 37°C for 2 h.

RPMI 1640 Complete Medium: 90% RPMI 1640 (Hyclone) + 9% FPS (Hyclone) + 1% penicillin/streptomycin (ScienCell).

### PBMCs stimulation with Mtb-derived antigens and cytokine quantification

At the final concentration of 10 μg/ml (Zhao et al., 2014) H37Rv HupB, BJ LAM, BCG LAM, H37Rv HtdY, early secreted antigenic target-6 kDa (ESAT-6) and Culture filtrate protein 10 (CFP10) antigen and 100 ng/ml Lipopolysaccharides (LPS) inducer were added to the cell culture plate. The four antigens H37Rv HupB, BJ LAM, BCG LAM, and H37Rv HtdY were extracted by our laboratory, EAST-6 and CFP-10 were purchased from Oxford Immunotec, and LBS was purchased from Hyclone. And the same volume of 1 × PBS was added to the control group. The cells were incubated for 24 h at 37°C with 5% CO_2_.

The cell growth was observed under an optical microscope. The cell culture medium was taken and centrifuged at 1500 rpm for 10 min in a 1.5 mL sterile EP tube. The cells and supernatant were collected (stored at −80°C). The detection of cytokines in the samples was measured by ELISA. ELISA kits for IL-6 and IFN-γ were purchased from Neobioscience (QuantiCyto^®^ Human IL-6 ELISA kit and QuantiCyto^®^ Human IFN-γ ELISA kit). The detection method shall be tested according to the kit instructions. SuPerMax 3000FA multifunctional enzyme label instrument purchased from Shanghai Flash Spectrum Biotechnology Co., LTD.

### Data processing and statistical analysis

The statistical analysis was performed using GraphPad Prism 8.0.2. Comparisons between the two groups were carried out using an unpaired two-tailed t-test and triple comparisons were statistically analyzed using one-way ANOVA, *P* < 0.05 was considered statistically significant. The differences in cytokine secretion induced by antigen HupB on PBMCs between the PTB group and nPTB groups and HC groups were analyzed.

The Receiver operating characteristic curve (ROC curve) was constructed using GraphPad 8.0.2, and negative/positive diagnoses were made for experimental samples by calculating the Youden index.

## Results

### Analysis of cytokines secretion from HupB-stimulated PBMCs

A total of 23 clinical blood samples were collected at Wuhan Jinyintan Hospital, 11 from PTB patients and 12 from HC volunteers, 18 of male and 5 of female. PBMCs were separated from the blood for analysis. The newly extracted cells were observed under an inverted microscope, and they were small in size, round and transparent, they were suspended in the medium. After 2 h of cultivation, the cells were evenly distributed in the culture hole, and part of the cells showed adherent growth, spherical, and a large number of cells.

The cytokine levels of IL-4, IL-12p70, TGF-β1, TNF-α, IFN-γ, IL-6, and IL-17A from untreated PBMCs supernatants were examined using ELISA, to get the baseline data of PTB patients and healthy people for further investigation.

**FIGURE 1:**
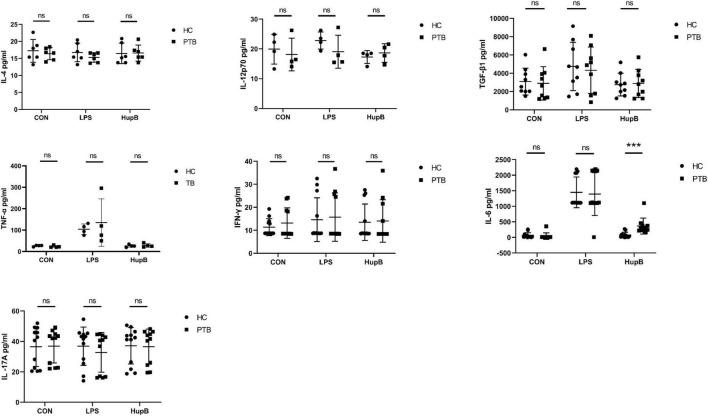
Secretion of PBMCs cytokines in samples before and after HupB induction. CON: Cytokines release in PBMCs isolated from healthy people and PTB patients. After 24 h culture *in vitro*, the secretion levels of IL-4, IL-12p70, TGF-β1, TNF-α, IFN-γ, IL-6, and IL-17A in PBMCs from PTB patients; HupB: HupB induced cytokines release in PBMCs isolated from healthy people and PTB patients. A total of 24 h after 10 μg/ml H37Rv HupB stimulation, the secretion levels of IL-4, IL-12p70, TGF-β1, TNF-α, IFN-γ, IL-6, and IL-17A in PBMC from PTB patients. LPS: LPS induced cytokines release in PBMCs isolated from healthy people and PTB patients. A total of 24 h after 10 μg/ml H37Rv HupB stimulation, the secretion levels of IL-4, IL-12p70, TGF-β1, TNF-α, IFN-γ, IL-6, and IL-17A in PBMC from PTB patients. PTB, tuberculosis group; HC, healthy group; LPS, positive control; ***p < 0.001; “ns” represents *P*-value > 0.05.

After 24 h culture *in vitro*, the secretion levels of IL-4, IL-12p70, TGF-β1, TNF-α, IFN-γ, IL-6, and IL-17A in PBMCs from PTB patients and healthy people were shown in Table 2 concretely. Shortly, no significant difference in these cytokines’ secretion was observed between PBMCs from PTB patients or healthy volunteers (Figure 1).

**TABLE 2:** The cytokine expression levels of PTB patients and healthy people with direct detection and HupB stimulated.

	Direct detection		HupB stimulated	
	PTB (pg/ml)	HC (pg/ml)	PTB (pg/ml)	HC (pg/ml)
IL-4	16.38 ± 1.79	17.23 ± 3.35	16.59 ± 2.36	16.46 ± 3.03
IL-12 P70	18.14 ± 5.48	19.91 ± 4.99	18.63 ± 3.07	17.30 ± 2.15
TGF-β1	2897 ± 1816	3084 ± 1468	2881 ± 1534	2758 ± 1239
TNF-α	23.36 ± 4.87	26.50 ± 3.39	135.48 ± 110.64	104.20 ± 24.61
IFN-γ	13.11 ± 6.64	13.88 ± 10.73	14.05 ± 9.27	13.50 ± 7.90
IL-6	42.92 ± 104.10	65.86 ± 88.23	266.1 ± 313.9	75.62 ± 90.36
IL-17A	36.89 ± 11.02	36.49 ± 12.87	36.54 ± 11.52	37.13 ± 0.17

Then, the cytokines secretion levels of IL-4, IL-12p70, TGF-β1, TNF-α, IFN-γ, IL-6, and IL-17A from HupB-stimulated PBMCs were examined to analyze whether the HupB-induced immune response was different between PBMCs from PTB patients or healthy people.

A total of 24 h after 10 μg/ml H37Rv HupB stimulation, the secretion levels of IL-4, IL-12p70, TGF-β1, TNF-α, IFN-γ, IL-6, and IL-17A in PBMCs from PTB patients and healthy people were shown in Table 3 concretely, respectively. For IL-4, IL-12p70, TGF-β1, TNF-α, IFN-γand IL-17A, no significant difference was observed between PTB and HC groups, but only the level of IL-6 in supernatants of PBMCs from PTB patients was significantly higher than healthy control. And LPS stimulation induced no remarkable difference in these nine cytokines released in PBMCs from the PTB or HC group (Figure 1).

**TABLE 3:** The result of combination of IL-6 release assay and IGRA.

	SP PTB		SN PTB		Combined diagnosis	
	HUPB	ESAT-6 + CFP10	HUPB	ESAT-6 + CFP10	SP PTB	SN PTB
Specificity	85.71%	82.14%	80.00%	96%	89.28%	100%
Sensitivity	94.44%	91.67%	77.78%	72.22%	100%	80.56%

### Hupb was the immunodominant antigen to stimulate IL-6 secretion in PBMCs from PTB patients

**FIGURE 2:**
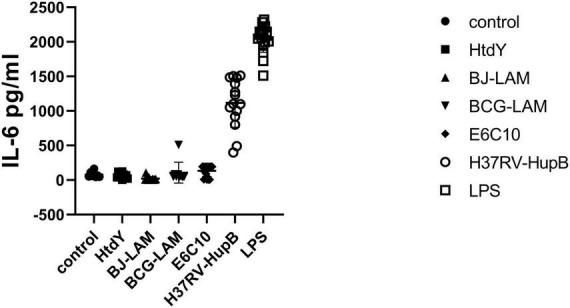
Different antigens stimulated secretion of IL-6 in PMBCs isolated from tuberculosis patients. A total of 64 blood samples from PTB patients were collected in Wuhan Jinyintan Hospital PBMCs were separated and then co-cultured with LPS, BJ-LAM, BCG-LAM, HtdY, ESAT-6, and CFP10, HupB or untreated control to induce IL-6 secretion. Control: Uninduced group.

To check if HupB was the only antigen to stimulate IL-6 secretion, a total of 64 blood samples from PTB patients were collected in Wuhan Jinyintan Hospital, PBMCs were separated and then co-cultured with BJ-LAM (*n* = 10), BCG-LAM (*n* = 9), HtdY (*n* = 10), ESAT-6 and CFP-10 (*n* = 10), HupB (*n* = 15) or untreated control (*n* = 10) to induce IL-6 secretion. The IL-6 levels in PBMCs supernatant of control, BJ-LAM treated, BCG-LAM treated, HtdY treated, ESAT-6 and CFP-10 treated, HupB treated were 20.94 ± 27.86 pg/ml, 106.7 ± 151.2 pg/ml, 66.52 ± 44.55 pg/ml, 134.6 ± 82.13 pg/ml, 799.8 ± 622.8 pg/ml, respectively. The IL-6 level of HupB-stimulated PBMCs was significantly higher than untreated control or BJ-LAM, BCG-LAM, HtdY, ESAT-6, and CFP-10-treated PBMCs. The results implied that only HupB stimulated IL-6 release in PBMCs from PTB patients (Figure 2).

### Single-center small sample clinical validation of HupB stimulated IL-6 releasing in PBMCs

To verify the effects of HupB-induced IL-6 secretion in PBMCs from PTB patients, 112 clinical blood samples (Male: 72, Female: 40) were collected from Wuhan Jinyintan hospital, 49 from PTB patients, 23 from nPTB patients, and 40 from HC.

In PBMCs collected from PTB patients, the baseline releasing level of IL-6 was 67.14 ± 132.10 pg/ml, HupB treatment significantly (^****^P < 0.0001) increased IL-6 secretion level to 843.10 ± 611.50 pg/ml. In PBMCs from nPTB patients, before and after HupB stimulation, the IL-6 secretion levels were 28.97 ± 28.46 pg/ml, and 50.17 ± 92.70 pg/ml, respectively. And in PBMCs from healthy volunteers, the IL-6 secretion levels before and after HupB treatment were 51.10 ± 55.99 pg/ml and 53.51 ± 57.04 pg/ml, respectively.

**FIGURE 3:**
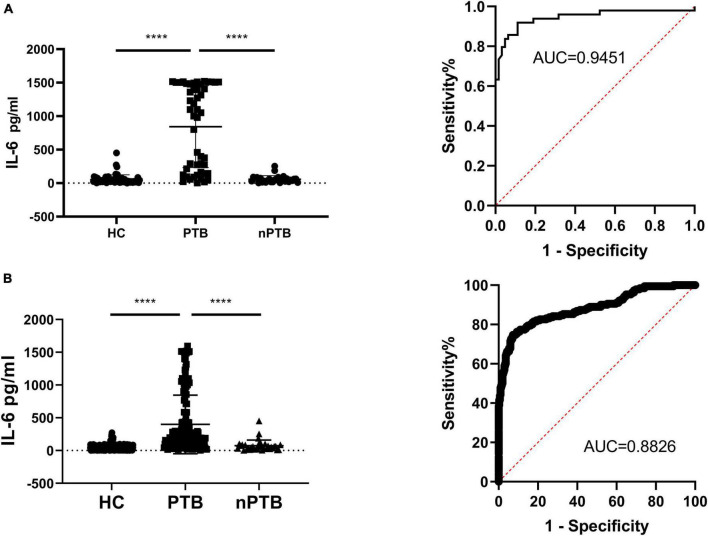
Secretion of IL-6 in PBMCs samples before and after HupB induction. **(A)** HupB induced IL-6 release in PBMCs isolated from healthy control (HC), non-tuberculous pulmonary disease patients (nPTB) and pulmonary tuberculosis patients (PTB), single center clinical validation; **(B)** HupB induced IL-6 release in PBMCs isolated from healthy control (HC), non-tuberculous pulmonary disease patients (nPTB) and pulmonary tuberculosis patients (PTB), multi-center clinical validation. PTB, tuberculosis group; nPTB, group; HC, healthy group; Control, uninduced control group; ****p < 0.0001.

Analysis of the ROC curve illustrated that the Area Under Curve (AUC) for PTB diagnosis was 0.9195, with a specificity of 83.67% and sensitivity of 90.48% when the recommended cut-off value of 101.6 pg/ml was applied. And false positive rate (FPR) was 9.52% and the false negative rate (FNR) was 16.33% (Figure 3A).

### Multi-center trial for HupB-induced IL-6 release assay

To furtherly verify the capability of HupB-induced IL-6 release assay for PTB diagnosis, 496 clinical blood samples were collected from 6 hospitals, 190 from PTB patients, 33 from nPTB patients, and 273 from HC, male for 292 and female for 204.

In the PTB group, the HupB-induced IL-6 secretion level was 490.5 ± 487.5 pg/ml, which was significantly (^****^p < 0.0001) higher than that in the nPTB group (73.53 ± 85.72 pg/ml) and HC group (45.25 ± 42.58 pg/ml).

Analysis of the ROC curve indicated that the AUC for PTB diagnosis was 0.8826 in the multi-center trial, with a specificity of 78.9% and sensitivity of 91.50% when the recommended cut-off value of 101.6 pg/ml was applied. And false positive rate (FPR) was 8.5% and the false negative rate (FNR) was 21.1% (Figure 3B).

### Comparison of HupB-induced IL-6 release assay and IGRA for TB diagnosis

To compare the effectiveness of HupB-induced IL-6 release assay and ESAT-6 and CFP-10-medicated IGRA for TB diagnosis, another 89 clinical samples were collected from Wuhan Jinyintan Hospital, Xiangyang city hospital for tuberculosis and Xiangyang Central Hospital, 28 smear-positive PTB patients, 25 of smear-negative PTB patients and 36 HC volunteers.

**FIGURE 4:**
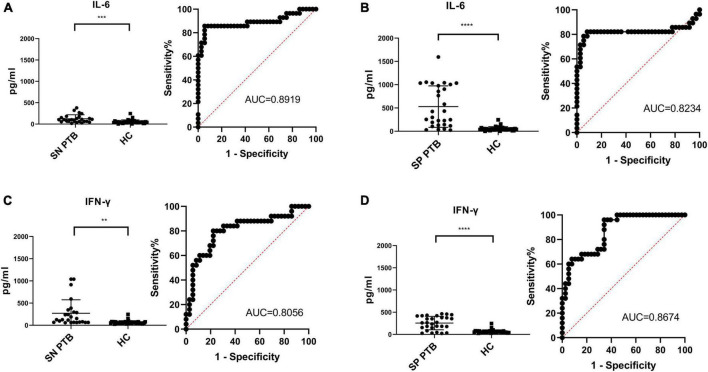
Comparison of HupB induced IL-6 release assay and IGRA for smear positive/negative PTB diagnosis. Diagnosis results of 25 of smear negative PTB patients with HupB **(A)** and E6C10 **(C)**; Diagnosis results of 28 of smear positive PTB patients with HupB **(B)** and E6C10 **(D)**. SP PTB, smear positive PTB group; SN PTB, smear negative PTB group; HC, healthy group; ****p < 0.0001; ***p < 0.001; **p < 0.01.

In smear-positive PTB patients, HupB stimulated PBMCs released IL-6 to a level of 377.8 ± 340.6 pg/ml, it was significantly (^****^p < 0.0001) higher than HupB treated PBMCs from the HC group, which was 53.88 ± 44.02 pg/ml; meanwhile, ESAT-6 and CFP10 treated PBMCs from smear-positive PTB patients secreted IFN-gamma to a level of 212.7 ± 161.2 pg/ml, which was significantly (^****^p < 0.0001) higher than that of HC group (63.92 ± 39.03 pg/ml). Analysis of the ROC curve of smear-positive PTB illustrated that the AUC for smear-positive PTB diagnosis was 0.8919 with a specificity of 85.71% and sensitivity of 94.44%. And FPR was 5.56%, FNR was 14.29% (Figure 4A). For ESAT-6 and CFP-10 mediated IGRA, the AUC for smear-positive PTB diagnosis was 0.8234 with a specificity of 82.14% and sensitivity of 91.67%. FPR was 8.33% and FNR was 17.86% (Figure 4B).

In smear-negative PTB patients, HupB-stimulated PBMCs released IL-6 to a level of 124.5 ± 92.26 pg/ml, which was significantly (^***^p < 0.001) higher than HupB-treated PBMCs from the HC group (as mentioned above, 53.88 ± 44.02 pg/ml). And ESAT-6 and CFP10 treated PBMCs from smear-negative PTB patients secreted significantly (^**^p < 0.01) higher IFN-gamma levels (351.0 ± 599.5 pg/ml) compared with the HC group. Analysis of the ROC curve illustrated that the AUC for smear-negative PTB diagnosis was 0.8056 with a specificity of 80.00% and sensitivity of 77.78%. And FPR was 22.22% and FNR was 20.00% (Figure 4C). For ESAT-6 and CFP-10 mediated IGRA, the AUC for smear-negative PTB diagnosis was 0.8778 with a specificity of 96.00% and sensitivity of 72.22%. FPR was 27.78% and FNR was 4.00% (Figure 4D).

### Combination of HupB-induced IL-6 release assay and IGRA for TB diagnosis

Interferon-γ release test has been shown in some clinical studies of tuberculosis diagnosis to have high sensitivity but relatively low specificity for IFN-γ. However, its operation is relatively complex and expensive, and it cannot distinguish between latent tuberculosis and active tuberculosis. This diagnostic method is limited for immunodeficient patients. According to the specific induction of IL-6 by PBMC of *Mycobacterium tuberculosis* HupB in patients with tuberculosis, in order to compare the detection effect of two antigens on patients with negative and positive bacteria, and analyze the application value of combined diagnosis.

The collected blood samples were separated by PBMC, and protein HupB and protein ESAT6-CFP-10 (E6C10) with a final concentration of 10 μg/ml were added to induce PBMC for 24 h. After induction, the cell culture supernatant was collected to measure the release of IL-6 in the supernatant, and the cells were stored for later use. When isolating PBMC, the sample should be discarded if the number of cells is small or the obvious cloud layer cannot be isolated.

Statistical analysis was performed on the release of IL-6 and IFN-γ induced by antigen E6C10 in PBMC of patients with bacterial-negative pulmonary tuberculosis, patients with bacterial-positive pulmonary tuberculosis and healthy volunteers to confirm whether there was any difference. The ROC survival curve was drawn, and the optimal cut-off value was obtained by calculating the Yoden index, and the effect of this method in the clinical diagnosis of pulmonary tuberculosis was evaluated.

The combination of IL-6 release assay and IGRA may have better sensitivity on TB diagnosis, we then analyzed the combined diagnosis and the results illustrated that in 28 smears-positive PTB patients, 22 (78.6%) were positive in both the IL-6 release assay and IGRA, another 2 (7.1%) were positive in HupB assay, and one (3.6%) was positive in IGRA assay; the specificity was 89.28% and the sensitivity was 100%. In 25 smears-negative PTB patients, 19 (76%) were positive in both the IL-6 release assay and IGRA, another 1 (4%) was positive in the HupB assay, and 5 (20%) was positive in the IGRA assay; the specificity was 100% and the sensitivity was 80.56% (Table 3).

The results suggested HupB induced IL-6 release assay can elevate IGRA-related diagnostic detection rate of TB. We have some highlights:

1. HupB can specifically stimulate PBMCs from tuberculosis patients to release IL-6.

2. HupB-induced release of IL-6 had potential clinical application value for TB diagnosis.

3. The sensitivity and specificity of the combined diagnosis of HupB-induced IL-6 release assay with ESAT-6 and CFP-10 induced IGRA was better than that of the single index, which can be used as potential auxiliary diagnostic markers.

## Discussion

Many antigens in the filtrate of *M. tuberculosis*, such as proteins and liposans, have unique structures and characteristic epitopes of antigens. Different antigen structures and epitopes activate different immune cell subsets, produce effector immune cells and memory immune cells, and induce different cytokine profiles. Due to the different structure and epitopes of hupB and other antigens (HTDY and LAM) such as E6C10, when HupB protein re-stimulates PBMC of tuberculosis patients *in vitro*, more memory immune cells producing IL-6 will be activated, prolifitated and produce IL-6. There were significant differences in IL10 produced when HTDY and LAM stimulated PBMC of tuberculosis patients again, and significant differences in IFN-γ produced when E6C10 stimulated PBMC of tuberculosis patients again.

HupB, also known as Rv2986c, is a histone-like protein that take part in iron metabolism and may play a role in host infection (Kalra et al., 2018). Recent studies suggested that HupB was a potential biomarker for TB diagnosis (Sivakolundu et al., 2013; Sritharan et al., 2015). The HupB gene was amplified in TB-carrying people (33832892) or animals (16272503) blood samples. Particular antigenic fragments (aa 63-161) or antibodies of HupB were identified in PTB patients (25037869) (Sritharan et al., 2015). However, nucleic acid or peptide of HupB may exist in Mtb carriers, it’s an arduous challenge to distinguish carrier and PTB patients by directly detecting HupB. Antibodies of HupB could be detected in cured PTB patients, limiting its usage in TB diagnosis. The cellular immunologic response of HupB could be a better choice for the diagnosis of active tuberculosis.

In the current study, we scanned the secreted cytokine spectrum of HupB-stimulated PBMCs from PTB patients, and found that IL-6 was the only boosted inflammatory factor, suggesting HupB-induced IL-6 releasing may be an immune marker of active tuberculosis. IL-6 was first identified in 1973 as a cytokine secreted from Th2 cells and macrophages and plays a crucial role in antibody production by B cells (Tsantikos et al., 2010; Tanaka et al., 2014; Taurone et al., 2015). Prati Pal Singh and Amit Goyal found that pathogenic Mtb-infected macrophages secreted the highest fold change of IL-6, compared with IL-1, IL-10, IL-12p40, and IL-12p70, implying that IL-6 was the only major cytokine elaborated by Mtb infected macrophages (Singh and Goyal, 2013). Serum IL-6 elevation was found in PTB patients, and the level of serum IL-6 was consistent with the progression or healing of tuberculosis (Zambuzi et al., 2016). These findings suggested that Mtb infection-induced IL-6 releasing may play important roles in host immune response and could be a potential marker for TB diagnosis. However, various infections could induce an IL-6 increase in blood, including SARS-Cov-2 and the specificity of serum IL-6 for TB diagnosis was unconvinced (Garbers et al., 2018). In the current study, we first revealed HupB incubating induced IL-6 releasing in PBMCs from PTB patients, with huge potential for active tuberculosis diagnosis, and constructed a multi-center trial to verify the effectiveness, and the results showed that HupB induced IL-6 release assay has better specificity and sensitivity compared with ESAT-6 and CFP-10 induced IGRA in smear-positive PTB, and better sensitivity in smear-negative PTB patients.

In this study, clinical samples were collected from six hospitals in Hubei and Henan provinces for clinical verification. To a certain extent, HupB protein of Mycobacterium tuberculosis can specifically induce the release of IL-6 from PBMC of patients with tuberculosis, and HupB can induce the release of IL-6 from macrophages. The results of this study provide reference for the subsequent research on the mechanism of interaction between Mycobacterium tuberculosis and immune cells, and provide a certain theoretical basis and potential application value for optimizing the clinical diagnosis of tuberculosis. The shortcomings of the study lie in that no volunteers with latent tuberculosis were recruited and the study site was not large enough. More samples are needed for further verification and analysis.

The immune response process in the body is very complex, and the immune response composed of a single immune cell is not enough to explain the whole immune process of tuberculosis. In addition to macrophages, a variety of immune cells, such as CD4 + T cells and B lymphocytes, can release IL-6 (Hadizadeh Tasbiti et al., 2016). Subsequent studies on the effect of HupB on the signaling pathway of macrophages can be further explored, as whether other immune cells can also release IL-6.

## Data availability statement

The raw data supporting the conclusions of this article will be made available by the authors, without undue reservation.

## Ethics statement

The studies involving human participants were reviewed and approved by written informed consent were acquired from all subjects, and subjects’ privacy is always respected. The investigation received approval from the Biomedical Ethics Committee of Wuhan Polytechnic University (Ratification number: 20190915004). The patients/participants provided their written informed consent to participate in this study.

## Author contributions

XY, HuW, and SZ conducted the study conception and design. SW collected samples, designed cohort, clinical validation, and analyzed the data. XY, SJ, and HuW contributed to interpreting the results and writing the manuscript. YL, HZ, YW, XL, YZ, PH, and HaW supervised the project. All authors read and approved the final manuscript.
