# Supplementary material for: Development of a new cellular immunological detection method for tuberculosis based on HupB protein induced IL-6 release test
Source: Front Microbiol. 2023 Apr 3;14:1148503. doi: 10.3389/fmicb.2023.1148503 (PMC10106748; doi:10.3389/fmicb.2023.1148503)
Supplement: Supplementary file 1 [file Data_sheet_1.DOCX]

**The Supplemental Material**

**1. Materials and methods**

**1.1 Experimental strain**

Escherichia coli DH5α and BL21 (DE3) were preserved by our laboratory; H37Rv standard strain was provided by Shanghai Jiaotong University School of Medicine；Recombinant plasmid pGEX-6p-1, provided by Wuhan Jinkairui Biological Engineering Co., Ltd.

**1.2 Prokaryotic expression and purification of HupB protein**

Based on the gene sequence of Mycobacterium tuberculosis H37Rv Rv2986, a recombinant plasmid pGEX-6p-1-Rv2986c was constructed; Transformed the recombinant plasmid into Escherichia coli DH5α and induce expression in BL21 (DE3). SDS-PAGE analyzed the expression of HupB protein in Escherichia coli; GST column purification of recombinant protein HupB; SDS-PAGE determination of protein molecular weight; Triton X-114 liquid phase separation to remove endotoxin; BCA method to detect protein concentration.

**1.3 Circular Dichroism Spectroscopy to Detect the Secondary Structure of Recombinant Protein HupB**

A JASCO J-1500 circular dichroism spectrometer was used to scan its far-ultraviolet circular dichroism (CD) spectrum.

**2. Results**

**2.1 Prokaryotic expression and purification of HupB protein**

The recombinant protein was purified by a GST column and detected by SDS-PAGE electrophoresis, and it was found that it had a band of interest at 51KDa (Fig.S1). The size of the band was the sum of the molecular weight of plasmid pGEX -6p-1 and HupB protein; Recombinant protein purity was 85%; Use Bandscan software to determine the purity of the target protein to be more than 90%.

The concentration of HupB protein after concentration was determined by Pierce BCA Protein Assay Kit method. During the determination, the protein standard curve was shown in Fig.S2. Substitute the absorbance of HupB protein into the standard curve regression equation, the calculated concentration of HupB protein was 853 µg/ml. After the endotoxin in the protein was removed by EtEraserTM HP endotoxin removal kit, the final endotoxin content in HupB protein was less than 0.3 EU/ml, which can be used for subsequent experiments.

**2.2 Circular Dichroism Spectroscopy to Detect the Secondary Structure of Recombinant Protein HupB**

The obtained HupB recombinant protein was scanned by circular dichrograph 5 times at 25°C and the average was taken. The circular dichroism result of HupB recombinant protein was shown in Fig.S3. The secondary structure elements of HupB recombinant protein at 25℃ were obtained by using the software that comes with the instrument. Among them, 13.4% α helix, 32.1% β sheet, and 54.5% random coils were basically consistent with the predicted HupB secondary structure. After protein detection, the content of nucleic acid and other impurities in the purified protein is very low, which can be used for subsequent cell experiments.

**Supplementary Figures**

**
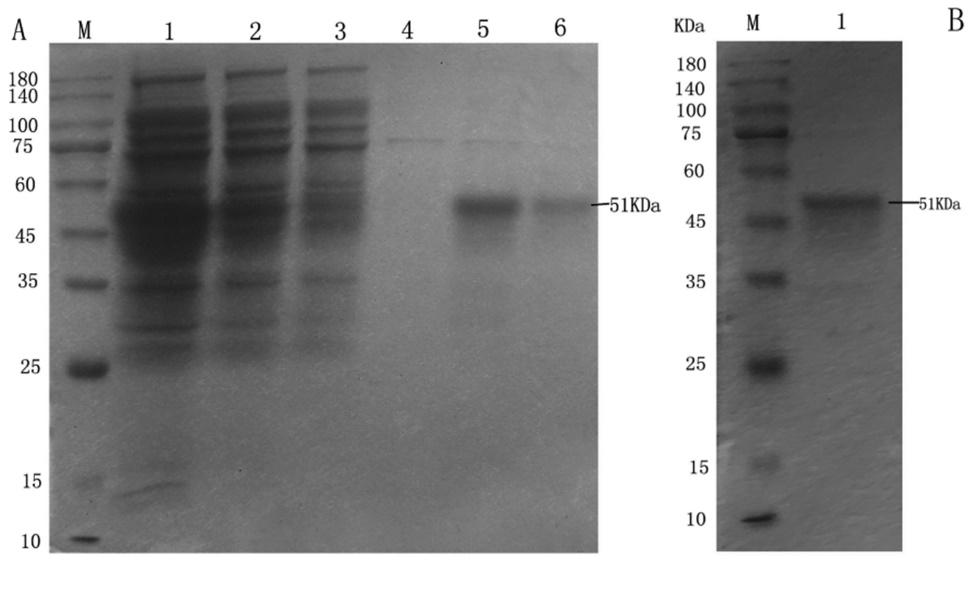
**

Figure S1 Purification results of HupB protein. Note: A: M, protein marker; 1, loading solution; 2, flow-through solution; 3, washing solution; 4, 5, 6, eluents; B: M, protein marker; 1, protein purification products.


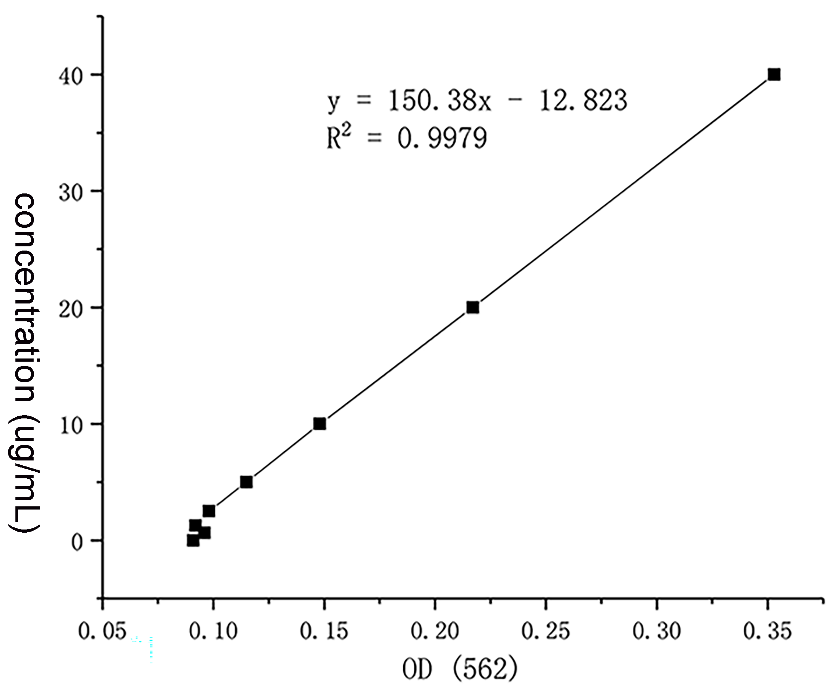


Figure S2 Protein standard curve. The concentration of HupB protein after concentration was determined by Pierce BCA Protein Assay Kit method. Protein standard curve during the determination.


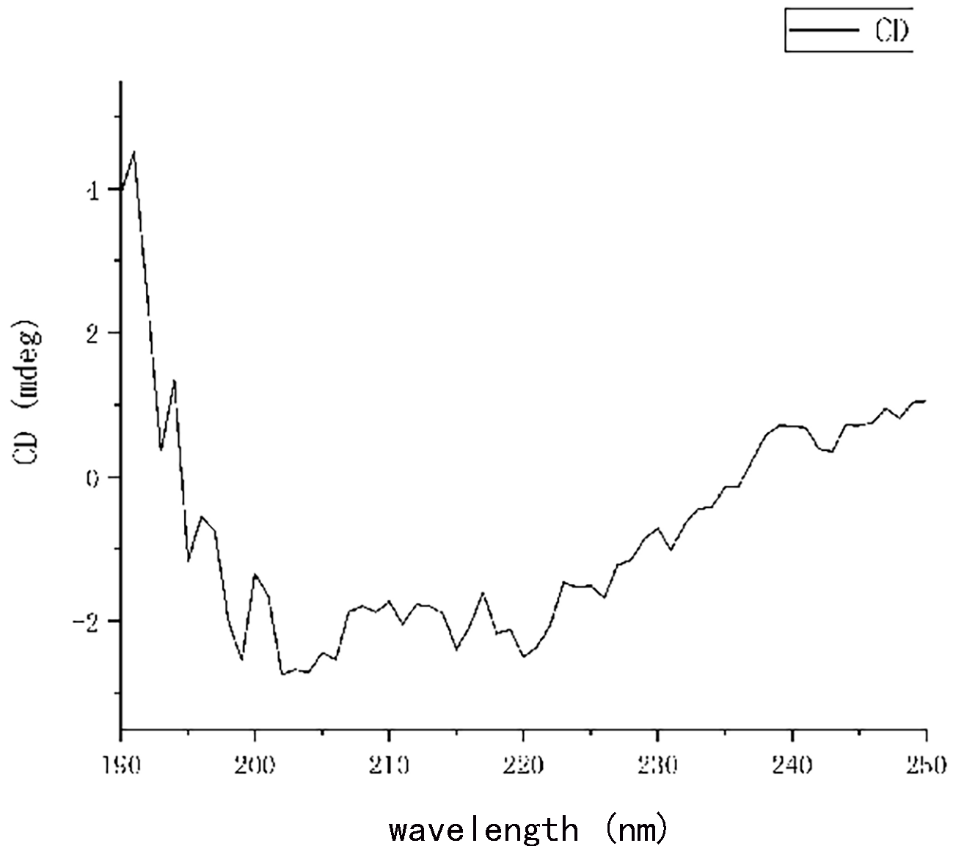


Figure S3 Circular dichroism results of recombinant protein HupB. The obtained HupB recombinant protein was scanned by circular dichrograph 5 times at 25℃ and the average was taken.
